# Universal skepticism of ChatGPT: a review of early literature on chat generative pre-trained transformer

**DOI:** 10.3389/fdata.2023.1224976

**Published:** 2023-08-23

**Authors:** Casey Watters, Michal K. Lemanski

**Affiliations:** ^1^Faculty of Law, Bond University, Gold Coast, QLD, Australia; ^2^Institute for Human Resource Management, WU Vienna, Vienna, Austria

**Keywords:** ChatGPT, large language model (LLM), transformer, GPT, disruptive technology, artificial intelligence, AI

## Abstract

ChatGPT, a new language model developed by OpenAI, has garnered significant attention in various fields since its release. This literature review provides an overview of early ChatGPT literature across multiple disciplines, exploring its applications, limitations, and ethical considerations. The review encompasses Scopus-indexed publications from November 2022 to April 2023 and includes 156 articles related to ChatGPT. The findings reveal a predominance of negative sentiment across disciplines, though subject-specific attitudes must be considered. The review highlights the implications of ChatGPT in many fields including healthcare, raising concerns about employment opportunities and ethical considerations. While ChatGPT holds promise for improved communication, further research is needed to address its capabilities and limitations. This literature review provides insights into early research on ChatGPT, informing future investigations and practical applications of chatbot technology, as well as development and usage of generative AI.

## 1. Introduction

ChatGPT, developed by OpenAI, is a new language model that has generated significant buzz within the technology industry and beyond. With the launch of artificial intelligence-based Chat Generative Pre-trained Transformer (ChatGPT), OpenAI has taken the academic community by storm, forcing scientists, editors and publishers of scientific journals to rethink and adjust their publication policies and strategies. Whereas availability of ChatGPT has been sanctioned in some jurisdictions (e.g., China, Italy), like the creation of the internet, the emergence of ChatGPT may possibly become a marking line of a new era, and scholars need to embrace this technological development. Since its release, researchers have been exploring its capabilities and limitations across various fields such as healthcare, business, psychology, and computer science, building on the research of earlier language models (Testoni et al., 2022; Rocca et al., 2023; Roy et al., 2023). This literature review aims to provide an overview of early ChatGPT literature in multiple disciplines, analyzing how it is being used and what implications this has for future research and practical applications.

The literature reviewed in this study includes a range of perspectives on ChatGPT, from its potential benefits and drawbacks to ethical considerations related to the technology (Seth et al., 2023). The findings suggest that while early research is still limited by the scope of available data, there are already some clear implications for future research and practical applications in various fields. For example, many scholars have raised concerns about ChatGPT's potential impact on employment opportunities across different industries (Qadir, 2022; Ai et al., 2023). While early studies suggest promising results for chatbot technology in healthcare settings, there are still significant ethical considerations (Rahimi and Talebi Bezmin Abadi, 2023) to be addressed before widespread implementation can occur.

ChatGPT uses advanced machine learning techniques to generate natural language responses, making it an attractive tool for various industries seeking more efficient communication with customers or clients. Its potential applications range from customer service chatbots to virtual assistants in healthcare settings (Sallam, 2023). However, as ChatGPT is still a relatively new technology, there are many questions about its capabilities and limitations that need to be addressed by researchers across different fields. This literature review aims to provide insights into how early research on ChatGPT has evolved, highlighting key findings from sentiment analysis of articles related to chatbot technology in various academic areas.

## 2. Methodology

While most of the discussion takes place in the media, in committee meetings or informal fora, we also see that systematic scholarly research has started to emerge rapidly. From the launch of ChatGPT in late November 2022 until April 2023 there were 154 publications, with only two publications released in 2022. While we appreciate all the research dedicated explicitly to new technologies, for quality assurance, we limit our review to sources included in the rigorously monitored Scopus database, and in this paper we report only on a review of Scopus-indexed publications.

The sentiment analysis conducted in two popular software packages using different dictionaries showed dominance of negative sentiment in all papers examined and across all disciplines. We however refrain from conclusions about a general negative sentiment, since words expressing attitudes are subject-specific. Therefore, we selected a sub-sample of papers in the three disciplines (using the Scopus classification) in which authors have completed both formal education and possess research experience: (1) economics, econometrics, and finance, (2) business, management, and accounting, and (3) social sciences, which we read paragraph by paragraph, assessing sentiment of each as positive, neutral, or negative.

When reviewing publications for this paper, we followed usual procedures recommended for literature reviews in new and emerging fields of research (Gancarczyk et al., 2022; Liang et al., 2022). Having set the scope of the research to only Scopus-indexed publications published between November 2022 and April 2023, we first identified papers which contain the name “ChatGPT” either in the title, abstract, or keywords. This resulted in 156 entries. Next, we sorted out the received pool of papers into 22 subject areas. One hundred forty publications fitted into the pre-established categories, while the remaining 16 were classified as multidisciplinary. For details on the distribution and actual publications, see Table 1.

**Table 1:** Early SCOPUS indexed publications on ChatGPT (through 8 April 2023).

**Subject area**	**Number of articles**	**Citations**
Medicine	64	Ahn, 2023; Alberts et al., 2023; Ali and Djalilian, 2023; Ali et al., 2023; Anderson et al., 2023; Ang et al., 2023; Berger and Schneider, 2023; Bernstein, 2023; Bhatia and Kulkarni, 2023; Bhattacharya et al., 2023; Borges, 2023; Boßelmann et al., 2023; Cascella et al., 2023; Cox, 2023; Curtis, 2023; Dahmen et al., 2023; D'Amico et al., 2023; DiGiorgio and Ehrenfeld, 2023; Donato et al., 2023; Doshi et al., 2023; Eardley, 2023; Elwood, 2023; Fijačko et al., 2023; Gordijn and Have, 2023; Hirosawa et al., 2023; Homolak, 2023; Huang et al., 2023; Huh, 2023b; Jungwirth and Haluza, 2023; Kahambing, 2023; Khan et al., 2023; Kim, 2023; Krettek, 2023; Lahat and Klang, 2023; Lecler et al., 2023; Lee, 2023b; Levin et al., 2023; Liebrenz et al., 2023; Looi, 2023; Macdonald et al., 2023; Maeker and Maeker-Poquet, 2023; Mann, 2023; Mogali, 2023; Moisset and Ciampi, 2023; Naumova, 2023; Nuryana and Pranolo, 2023; Ollivier et al., 2023; Park et al., 2023; Patel and Lam, 2023; Paul et al., 2023; Potapenko et al., 2023; Prada et al., 2023; Quintans-Júnior et al., 2023; Rozencwajg and Kantor, 2023; Sallam, 2023; Salvagno et al., 2023; Schorrlepp and Patzer, 2023; Šlapeta, 2023; Strunga et al., 2023; Temsah et al., 2023; The Lancet Digital Health, 2023; Yadava, 2023
Social Sciences	56	Abdel-Messih and Kamel Boulos, 2023; Adetayo, 2023; Arif et al., 2023; Castro Nascimento and Pimentel, 2023; Chen, 2023; Choi et al., 2023; Cooper, 2023; Costello, 2023; Cotton et al., 2023; Cox and Tzoc, 2023; Crawford et al., 2023; Dasborough, 2023; Dwivedi et al., 2023; Emenike and Emenike, 2023; Eysenbach, 2023; Fergus et al., 2023; Fernandez, 2023; Gašević et al., 2023; Gilson et al., 2023; Gordijn and Have, 2023; Gregorcic and Pendrill, 2023; Haman and Školník, 2023; Harder, 2023; Hu, 2023; Huh, 2023a,b,c; Humphry and Fuller, 2023; Iskender, 2023; Johinke et al., 2023; Karaali, 2023; Kasneci et al., 2023; Lee, 2023b; Lim et al., 2023; Lin et al., 2023; Lund and Wang, 2023; Lund et al., 2023; Masters, 2023a,b; Morreel et al., 2023; Nautiyal et al., 2023; O'Connor, 2023; Panda and Kaur, 2023; Pavlik, 2023; Perkins, 2023; Rospigliosi, 2023; Schijven and Kikkawa, 2023; Siegerink et al., 2023; Strunga et al., 2023; Subramani et al., 2023; Tang, 2023; Teixeira da Silva, 2023; Tlili et al., 2023; Tsigaris and Teixeira da Silva, 2023; Yeo-Teh and Tang, 2023
Computer Science	25	Adetayo, 2023; Aljanabi et al., 2023; Budler et al., 2023; Cascella et al., 2023; Castro Nascimento and Pimentel, 2023; DiGiorgio and Ehrenfeld, 2023; Du et al., 2023; Dwivedi et al., 2023; Fernandez, 2023; Gao et al., 2023; Gašević et al., 2023; Haluza and Jungwirth, 2023; Lin et al., 2023; Lund and Wang, 2023; Lund et al., 2023; Mijwil et al., 2023; Panda and Kaur, 2023; Rospigliosi, 2023; Schijven and Kikkawa, 2023; Taecharungroj, 2023; Teubner et al., 2023; Thurzo et al., 2023; Tlili et al., 2023; Wang et al., 2023; Zhou et al., 2023
Multidisciplinary	16	Graham, 2022, 2023a,b; Stokel-Walker, 2022, 2023; An et al., 2023; Else, 2023; Lahat et al., 2023; Owens, 2023; Seghier, 2023; Stokel-Walker and Van Noorden, 2023; Thorp, 2023; Tools such as ChatGPT threaten transparent science; here are our ground rules for their use, 2023; Tregoning, 2023; van Dis et al., 2023; Wang S. H., 2023
Health Professions	14	Ali et al., 2023; Anderson et al., 2023; Cascella et al., 2023; DiGiorgio and Ehrenfeld, 2023; Huh, 2023a,c; Lecler et al., 2023; Lee, 2023a; Liebrenz et al., 2023; Patel and Lam, 2023; Sallam, 2023; Strunga et al., 2023; The Lancet Digital Health, 2023; Thurzo et al., 2023
Nursing	14	Ahn, 2023; Choi et al., 2023; Doshi et al., 2023; Fijačko et al., 2023; Gunawan, 2023; Harder, 2023; O'Connor, 2023; Odom-Forren, 2023; Sallam, 2023; Scerri and Morin, 2023; Siegerink et al., 2023; Strunga et al., 2023; Teixeira da Silva, 2023; Thomas, 2023
Engineering	11	Biswas, 2023a,b; Cooper, 2023; Du et al., 2023; Gao et al., 2023; Haluza and Jungwirth, 2023; Huang et al., 2023; Lin et al., 2023; Tong and Zhang, 2023; Wang et al., 2023; Zhou et al., 2023
Decision Sciences	10	Ali et al., 2023; Anders, 2023; Chatterjee and Dethlefs, 2023; Dwivedi et al., 2023; Elali and Rachid, 2023; Jungwirth and Haluza, 2023; Liebrenz et al., 2023; Lund et al., 2023; Patel and Lam, 2023; The Lancet Digital Health, 2023
Business, Management and Accounting	9	Ameen et al., 2023; Dasborough, 2023; Dwivedi et al., 2023; Iskender, 2023; Lim et al., 2023; Nautiyal et al., 2023; Paul et al., 2023; Short and Short, 2023; Taecharungroj, 2023
Psychology	8	Berger and Schneider, 2023; Bhatia and Kulkarni, 2023; Dasborough, 2023; Kahambing, 2023; Kasneci et al., 2023; Nuryana and Pranolo, 2023; Paul et al., 2023; Thurzo et al., 2023
Biochemistry, Genetics and Molecular Biology	7	Borges, 2023; Cahan and Treutlein, 2023; Hallsworth et al., 2023; Holzinger et al., 2023; Subramani et al., 2023; Tong and Zhang, 2023; Will ChatGPT transform healthcare?, 2023
Chemistry	6	Castro Nascimento and Pimentel, 2023; Emenike and Emenike, 2023; Fergus et al., 2023; Humphry and Fuller, 2023; Rillig et al., 2023; Zhu et al., 2023
Environmental Science	6	Halloran et al., 2023; Hirosawa et al., 2023; Jungwirth and Haluza, 2023; Lin et al., 2023; Rillig et al., 2023; Zhu et al., 2023
Mathematics	6	Du et al., 2023; Gao et al., 2023; Haluza and Jungwirth, 2023; Harder, 2023; Karaali, 2023; Wang et al., 2023
Immunology and Microbiology	5	Hallsworth et al., 2023; Quintans-Júnior et al., 2023; Šlapeta, 2023; Temsah et al., 2023; Tong and Zhang, 2023
Chemical Engineering	4	Castro Nascimento and Pimentel, 2023; Hallsworth et al., 2023; Holzinger et al., 2023; Huang et al., 2023
Economics, Econometrics and Finance	3	Ai et al., 2023; Dowling and Lucey, 2023; Paul et al., 2023
Neuroscience	3	Boßelmann et al., 2023; Graf and Bernardi, 2023; Moisset and Ciampi, 2023
Physics and Astronomy	3	Gregorcic and Pendrill, 2023; Karimabadi et al., 2023; Wang J., 2023
Arts and Humanities	2	Costello, 2023; Floridi, 2023
Agricultural and Biological Sciences	1	Borges, 2023
Dentistry	1	Sardana et al., 2023
Energy	1	Lin et al., 2023

## 3. Limitations

Inevitably, given the time scope of our review, the research reviewed here is all based on the 3rd version of ChatGPT and its various iterations. Version 4, released in mid-March 2023, offers considerable amendments, for instance accepts image input, and is capable of generating longer texts (Bhattacharya et al., 2023). Even though the fundamental assumptions and the basis on which ChatGPT works remains comparable, the greater variety of usage will lead to more profound impact on the work of scholars and what scientific institutions can achieve, as well as on recipients of academic research. Consequently, we expect fast emergence of further research on ChatGPT, and this review should serve only as a record of initial reactions in scholarly literature.

## 4. Discussion

The early research on ChatGPT suggests a range of potential benefits and drawbacks across various fields such as healthcare, business, psychology, and computer science, among others. Like the beginnings of the internet or the creation of digital assets (Lawuobahsumo et al., 2022; Kapengut and Mizrach, 2023; Watters, 2023), ChatGPT and its underlying technology have the opportunity for both positive and negative disruption. While many scholars have raised concerns about the impact of ChatGPT on employment opportunities in different industries, there are also significant ethical considerations to be addressed before widespread implementation can occur.

The negative sentiment expressed in the literature toward ChatGPT is noteworthy, as it suggests that there are concerns or challenges associated with using this technology in various fields. While some studies have highlighted the potential benefits of ChatGPT, such as its ability to generate human-like responses and improve user experience, others have raised ethical and practical issues related to privacy, bias, transparency, and accountability. For instance, some researchers have argued that although OpenAI pays special attention to eliminate abusive vocabulary and hate-speech by design, the generative AI tools trained on text from the open Internet may still perpetuate or even amplify existing biases in language use and data representation, leading to discriminatory outcomes for certain groups of people (e.g., people who do not classify into a binary gender classification, or ethnic minorities). While important for language models, this issue has overlap with concerns surrounding social media and other sources of information (Thornhill et al., 2019; Kurpicz-Briki and Leoni, 2021), the impact on policy making (Lamba et al., 2021), and the risk of fake news (Wu and Liu, 2018; Shu et al., 2019). Others have pointed out the limitations of current models in terms of their ability to handle complex social interactions, emotional expressions, and cultural nuances that are essential for effective communication with humans. Therefore, it is essential to ensure that chatbot technology is trained on diverse datasets that represent different demographics and cultures. Additionally, privacy concerns arise when personal information is collected by ChatGPT during conversations with users. It is crucial to establish clear guidelines for data collection and usage to protect user privacy. Furthermore, transparency and accountability are essential in chatbot technology to ensure that users understand how their data is being used and who has access to it. As researchers continue to explore this new technology, it will be important to consider both the benefits and drawbacks of chatbot technology to fully understand its implications for future research and practical applications.

Early research on ChatGPT suggests that while there are clear implications for future research and practical applications in various fields, further studies need to be conducted to fully understand its capabilities and limitations. This includes addressing ethical considerations such as privacy concerns and bias in data sets used by ChatGPT. Despite the potential benefits of chatbot technology, early research is still limited by the scope of available data. However, as ChatGPT continues to evolve and become more advanced, it has the potential to revolutionize communication across various industries. For instance, customer service chatbots can provide 24/7 support to customers, reducing wait times and improving overall satisfaction. While one might expect a positive reception of transformative technologies in the academic literature, the negative sentiment in the early literature may be explained by the types of literature. Approximately 12% of articles had ethics as a key word and just over 8% had plagiarism. Not only is it logical that addressing ethical issues would produce articles with a negative sentiment, but these articles may also be published faster.

There is an increasing number of articles using LLMs and other AI-based solutions to benchmark hypothetical physical theories (Adesso, 2023), to process data, or for integration into medical practice. However, these studies usually take more time to conduct and, in the case of those involving humans or animals, have additional delays in receiving research ethics approval. Despite medicine being the largest category, the majority of articles were theoretical and discussed possible applications of ChatGPT. Necessarily, these types of articles address potential problems, whereas later scientific articles may focus more on solutions and therefore show a more positive sentiment. Not only is it logical that new technology would be treated with skepticism in the academic world, but it perhaps should not be surprising that early literature addresses the ethical concerns of researchers and postulates the problems that will need to be addressed in future research.

In healthcare settings, virtual assistants can help patients schedule appointments (Chow et al., 2023), answer medical questions, and even monitor vital signs. Use of AI in the medical context has also been a focus of literature even outside on context of ChatGPT (Merhbene et al., 2022). However, the limitations of current models in terms of their ability to handle complex social interactions, emotional expressions, and cultural nuances that are essential for effective communication with humans need to be addressed before widespread implementation can occur. The literature suggests that the technology is not yet ready for clinical use, due to its limited ability and privacy issues (Au Yeung et al., 2023; De Angelis et al., 2023) and legal concerns (Dave et al., 2023). As researchers continue to explore this new technology, it will be important to consider both the benefits and drawbacks of chatbot technology in order to fully understand its implications for future research and practical applications.

There is a notable lack of legal scholarship addressing ChatGPT and large language models which is surprising considering legal considerations are addressed in many of the articles. This, however, may be explained by SCOPUS's lack of legal coverage. Law articles tend to have low citation rates as they cite the law itself more than other articles and may be local in nature (Eisenberg and Wells, 1998). Therefore, aside from law and society topics that recieve higher citations rates, legal scholarship is largely ignored by SCOPUS and the Web of Science databases. Nevertheless, in additional to ethical issues such as plagiarism, legal issues including intellectual property rights are often discussed (D'Amico et al., 2023). Intellectual property is perhaps a greater issue with AI creating visual art than with most outputs for LLMs, especially if the LLM is trained on a sufficiently large dataset. Additionally, ensuring accuracy is arguably even a larger risk than plagiarism. D'Amico et al. (2023) state that “ChatGPT had been listed as the first author of four papers, without considering the possibility of ‘involuntary plagiarism' or intellectual property issues surrounding the output of the model.” The approach taken by these papers (ChatGPT Generative Pre-trained Transformer and Zhavoronkov, 2022; O'Connor, 2022; King and ChatGPT, 2023) is understandable considering it is unknown what standards will be adopted in the future. However, using the output is not all that different from pulling from one's own knowledge. Academics must cite all sources of information not only for ethical reasons but also because it strengthens the claims of a paper. Not only does a failure of cite a source of information constitute plagiarism, but it weakens the paper. However, over time people learn and they may make statements without remember the original source. Thus, as something becomes common knowledge the source becomes less likely to be cited. When using LLMs, if something is outside of the knowledge of an author, they will need to look it up and in so doing will be ethically compelled to cite the source confirming the knowledge. We therefore argue that the primary danger is that authors will publish material produced by an LLM without ensuring its accuracy. It is not unethical to use an LLM, but authors must ensure the veracity of the final work regardless of whether they use an LLM like ChatGPT, the built-in spelling and grammar checking software, other in text editing software like MS Word, or other AI solutions to assist with writing. More research, therefore, should focus on the risk of fake resources, including journals publishing articles falsely purporting to be from famous academics, a problem that will undoubtedly increase with the proliferation of LLM technology.

**Figure 1:**
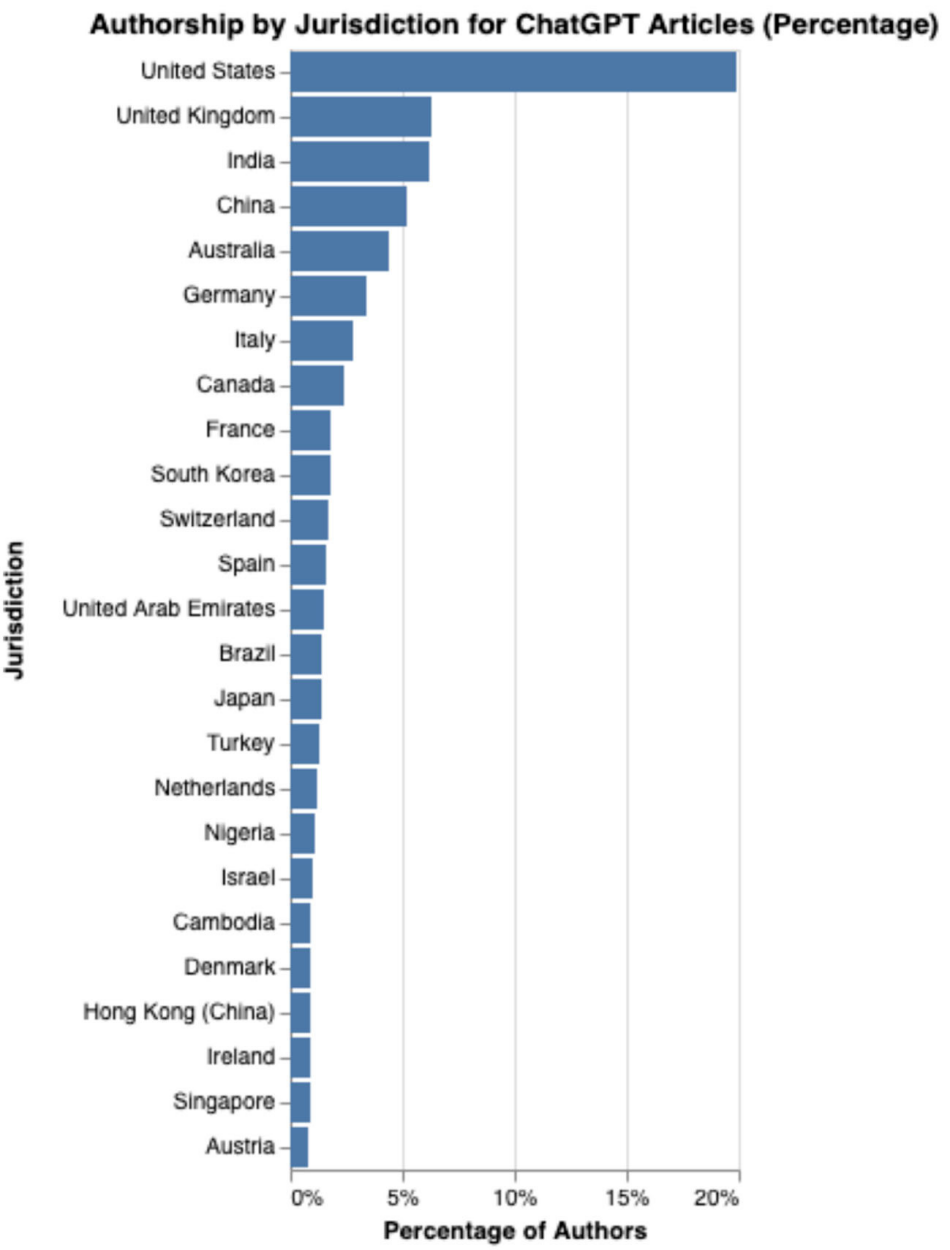
Authorship by country.

One surprising factor was the geographic universality of the findings. As can be seen in Figure 1, the top 25 countries by authorship included all six inhabited continents. In fact, the top three countries, the United States, United Kingdom, and India, are each on different continents. While there is a stronger representation of English-speaking countries, mainland China ranks fourth in the number of authors. It is perhaps not surprising that despite not being English speaking countries, China, Japan and South Korea, all leaders in technological development, would be amongst the top 15 regions.

Overall, the early literature on ChatGPT suggests that while it has great potential for improving communication across various industries, there are still many questions to be answered before its full impact can be realized. As researchers continue to explore this new technology, it will be important to consider both the benefits and drawbacks of chatbot technology to fully understand its implications for future research and practical applications. For instance, while ChatGPT has the potential to improve customer service by providing quick responses to frequently asked questions, there is a risk that customers may become frustrated if they encounter complex issues that cannot be resolved through automation. Additionally, chatbot technology may not be suitable for all industries or contexts, and it will be important to identify which applications are most effective in different settings. As ChatGPT continues to evolve and become more advanced, researchers must remain vigilant about the ethical considerations associated with its use, including privacy (Masters, 2023a) concerns, bias in data sets used by chatbots (Thornhill et al., 2019), transparency, accountability, and cultural sensitivity. By addressing these issues head-on, we can ensure that ChatGPT and similar solutions are deployed responsibly and effectively and the fact that all disciplines show negative sentiment toward ChatGPT in the early literature implies scholars are embracing this cautious approach.

## 5. Conclusion

In conclusion, the early literature on ChatGPT suggests that while there are promising results for its potential applications in various fields, there are also significant ethical considerations to be addressed before widespread implementation can occur. The negative sentiment across all academic areas related to early ChatGPT literature may be explained by limitations in current research or ethical concerns related to the use of GPT technology. As ChatGPT is still a relatively new technology, there are many questions about its capabilities and limitations that need to be addressed by researchers across different fields. The geographical dispersion and standing in university ranking of authors' institutions signals the interest is global in scope and a matter of importance for all sorts of institutions. In addition, the lack of comprehensive studies or datasets that can provide more nuanced insights into its capabilities and limitations beyond simple language processing tasks may contribute to negative sentiment across different disciplines. Overall, while early research is still limited by the scope of available data, there are already some clear implications for future research and practical applications in various fields. As ChatGPT technology continues to evolve, it will be important for researchers and stakeholders to work together to address these ethical considerations and ensure that this powerful tool is used responsibly and effectively across different industries.

## Author contributions

All authors listed have made a substantial, direct, and intellectual contribution to the work and approved it for publication.
